# Transmuscular quadratus lumborum block for postoperative pain and recovery after laparoscopic adrenalectomy: a randomized controlled trial

**DOI:** 10.1186/s12871-021-01494-4

**Published:** 2021-11-09

**Authors:** Qing Yuan, Sufang Lu, Xulei Cui, Yuelun Zhang, Yi Xie, Yushi Zhang, Weigang Yan, Zhigang Ji, Yuguang Huang

**Affiliations:** 1 Department of Anesthesiology, Peking Union Medical College Hospital, Chinese Academy of Medical Sciences & Peking Union Medical College, 100730 Beijing, China; 2 Medical Research Center, Peking Union Medical College Hospital, Chinese Academy of Medical Sciences & Peking Union Medical College, 100730 Beijing, China; 3 Department of Urology, Peking Union Medical College Hospital, Chinese Academy of Medical Sciences & Peking Union Medical College, 100730 Beijing, China

**Keywords:** Transmuscular quadratus lumborum block, Laparoscopic adrenalectomy, Pain

## Abstract

**Background:**

To investigate the role of transmuscular quadratus lumborum block (TMQLB) for postoperative pain control, patient satisfaction and recovery in laparoscopic adrenalectomy.

**Methods:**

Seventy-two patients aged between 18 and 70 years with an ASA I-II and scheduled for laparoscopic adrenalectomy were randomized to receive a single-shot TMQLB with 0.4 ml/kg 0.5 % ropivacaine or 0.4 ml/kg 0.9 % saline as placebo. The primary endpoint was pain on movement at 12 h after surgery evaluated by the numeric rating scale (NRS, 0–10). *P-*values < 0.05 was considered statistically significant. The secondary outcomes included pain at rest and pain on movement evaluated by the NRS, and postoperative recovery related parameters.

**Results:**

NRS on movement at 12 h after surgery was lower in the TMQLB group compared with the control (median 2 vs. 3, *p* = 0.024). Intraoperative fentanyl consumption was lower in the TMQLB group (247.08 ± 63.54 vs. 285.44 ± 74.70, *p* = 0.022). The rate of using postoperative rescue tramadol was also lower in the TMQLB group (5.6 vs. 27.8 %, *p* = 0.027). Similar incidences of nausea and vomiting were observed (11.1 vs. 25 %, *p* = 0.220). Patient satisfaction of pain service was better in the TMQLB group (83.3 vs. 25 %, *p* < 0.001) with shorter time to ambulation (16.5 vs. 21 h, *p* = 0.004) and flatus (18.5 vs. 23.5 h, *p* = 0.006).

**Conclusions:**

TMQLB showed better control of postoperative pain on movement for laparoscopic adrenalectomy with improved patients’ satisfaction of anesthesia, shorter time to ambulation and flatus.

**Trial registration:**

This study was registered at Clinicaltrials.gov (NCT03942237; registration date: 08/05/2019; enrollment date: 10/05/2019).

## Background

Laparoscopic adrenalectomy is considered as the gold standard treatment for adrenal lesions [1, 2]. Though minimally invasive, it still has postoperative pain associated with surgical incisions, pneumoperitoneum and surgical manipulations, which could increase the incidence of postoperative complications, decrease patient satisfaction, and prolong recovery. Regional block is an important element of multimodal analgesia that could reduce the need for opioids, mitigate stress response and enhance postoperative recovery [3].

**Fig. 1:**
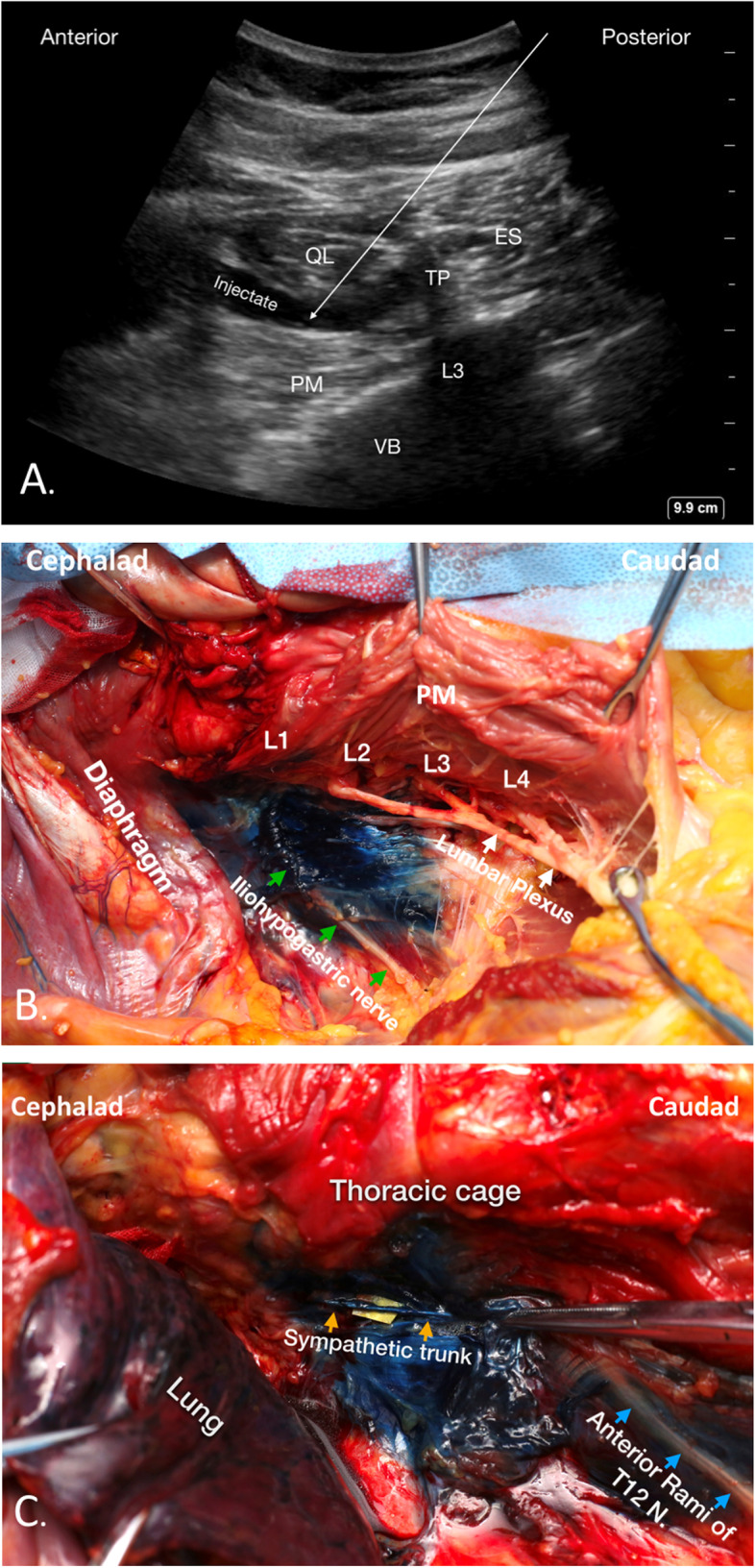
Ultrasound image showing TMQLB and the spread of dye. **A** Ultrasound image of TMQLB. The white arrow indicates the needle trajectory to the TMQLB at the L3 level, with the endpoint in the plane between the QL and PM muscles, which avoids piercing of the PM. The hypoechoic injectate spreads between the QL and PM muscles. **B** In the abdominal cavity, the dye (blue) is visualized surrounding the iliohypogastric nerve (green arrows), with no dye around the lumbar plexus (white arrows). **C** Within the thoracic cage, the thoracic paravertebral space is dissected. Dye (blue) can be visualized within the thoracic paravertebral space surrounding the thoracic sympathetic trunk (yellow arrows) and the segmental nerves (blue arrows). ES, erector spinae; PM, psoas muscle; QL, quadratus lumborum; TMQLB, transmuscular quadratus lumborum block; TP, transverse process; VB, vertebral body

Transmuscular quadratus lumborum block (TMQLB), first proposed by Børglum et al. [4] in 2013, is an emerging nerve block technique where local anesthetic is deposited in the fascial plane between psoas major (PM) and quadratus lumborum (QL) muscle. Anatomic evidence showed that the local anesthetic could spread along the thoracolumbar fascia to the thoracic paravertebral space, thus infiltrating thoracic spinal nerves and sympathetic trunk to provide both somatic and visceral analgesia for abdominal surgery [5]. We performed a TMQLB pilot study using a fresh cadaver (Fig. 1 A; unpublished data), obtaining results similar to those described by Dam et al. [5]. Specifically, methylene blue injected during TMQLB spread into the thoracic paravertebral space, staining the thoracic nerve (T10–T12) and thoracic sympathetic trunk, while no staining of the lumbar plexus was observed (Fig. 1B C).

Recent randomized controlled trials (RCTs) have demonstrated that TMQLB could be utilized in intraperitoneal abdominal surgeries, while its clinical use in retroperitoneal procedures has not been thoroughly investigated [6–8]. Therefore, the primary aim of our study is to investigate the analgesic efficacy of TMQLB in laparoscopic adrenalectomy. Our hypothesis is that a preoperative, single-shot TMQLB is effective in providing postoperative analgesia and facilitating recovery after laparoscopic adrenalectomy.

## Methods

### Study design

This study was designed with two stages after the Institutional Review Board approval (ZS-1559) of Peking Union Medical College Hospital, Beijing, China (Chairperson Prof Huizhu Zhao) on April 24, 2018, in accordance with principles of the Declaration of Helsinki (1964) and its subsequent amendments. Written informed consent form was obtained from all participants.

Since there were no previous studies regarding the efficacy of TMQLB for patients undergoing laparoscopic adrenalectomy, a pilot single arm study was first performed at Peking Union Medical College Hospital in January 2019. Ten patients scheduled for laparoscopic adrenalectomy, selected as per criteria described below, received TMQLB and other patients admitted in the same time period were used as control (n = 8). The pain on movement at 12 h after surgery, evaluated by numeric rating scale (NRS), was 2.30 ± 1.89 and 3.86 ± 2.25, respectively. Based on this observation, we estimated that a sample size of 33 would allow us to detect significant difference in terms of NRS on movement at 12 h after surgery with 80 % power (two-sided α = 0.05). Considering potential dropouts, we determined our sample size to be 36 per study group. Thereafter, a single center, prospective, randomized controlled, triple-blind trial was performed between May 2019 and September 2019. This trial was registered at ClinicalTrials.gov (NCT03942237; registration date: 08/05/2019; enrollment date: 10/05/2019).

### Patients

Eligible patients for this trial were those who between 18 and 70 years with ASA I-III and underwent laparoscopic adrenalectomy. Exclusion criteria were a known allergy to anesthetic medications, coagulopathy or on anticoagulants, chronic opioid therapy or history of substance abuse, enrolled in another trial, inability to properly describe postoperative pain to investigators (e.g., language barrier, neuropsychiatric disorder).

### Randomization and blinding

Eligible patients were randomized to TMQLB or control group with a ratio of 1:1 using the computerized SPSS package (version 22; SPSS Inc, Chicago, IL, USA). The randomization sequence was computer-generated by a professional statistician, who was not involved in the implementation and statistical analysis of the study. Allocation concealment was ensured by sealed, opaque, sequentially numbered envelopes. These assignment envelopes were opened after the inclusion of the patient in the study. The drugs were prepared by a nurse not involved in the study. The regional blocks were conducted by a single anesthesiologist and surgeries were performed by the same surgical team with a standardized retroperitoneal approach, who were blinded to patient allocation. A blinded observer recorded the study data.

### Intervention

Intravenous access and standard monitoring were established after the participant arrived at the operating room. The ultrasound-guided block was performed by the same experienced attending anesthesiologists.

In the TMQLB group, participant received a single-shot TMQLB with transmuscular approach at the surgical side. The patient was placed in the lateral position. A curved (C1-5) low-frequency probe of Philip CX 50 Ultrasound Scanner was positioned vertical to the iliac crest at the posterior axillary line. After the Shamrock sign was identified with ultrasound guidance, a 22-gauge needle (Pajunk Sonolong; GmbH Medizintechnologie, Geisingen, Germany) was inserted in plane and directed to the QL muscle under sterile conditions with local anesthetic infiltration. After proper positioning of the needle tip between PM and QL muscle was verified, 0.4 ml/kg 0.5 % ropivacaine was injected into the interfascial plane. In the control group, the block process was the same except that ropavacaine was substituted with 0.9 % saline.

The laparoscopic adrenalectomy was performed using the transabdominal technique. The patient is placed on beanbags in the lateral decubitus position. Three trocars are placed in the subcostal area. No local anesthesia was used during the surgical procedure.

### Anesthesia and analgesic regimen

After the ultrasound-guided block, the anesthesia induction regimen was as follows: propofol (2 mg/kg), fentanyl (1 ug/kg), and rocuronium (0.9 mg/kg). All patients received endotracheal intubation. For anesthesia maintenance, sevoflurane and a mixture of O2/N2O were used to keep the bispectral index (BIS) within 40–60. Fentanyl was administered as needed to control the heart rate and blood pressure within baseline ± 20 %. All patients received 4 mg ondansetron and 5 mg dexamethasone for prophylactic antiemetic treatment. For postoperative analgesia, patients received intravenous parecoxib 40 mg iv as rescue analgesia in case of NRS between 3 and 4, and tramadol 100 mg iv as rescue analgesia if NRS > 4.

### Study endpoints

The primary endpoint of this RCT was pain on movement at 12 h after surgery evaluated by the NRS (range, 0–10).

Secondary endpoints included:

1. Intraoperative fentanyl consumption.
2. Postoperative use of rescue analgesics.
3. Pain at rest evaluated by the NRS at 2, 4, 8, 12, 24, 48 and 72 h after surgery.
4. Pain on movement evaluated by the NRS at 2, 4, 8, 24, 48 and 72 h after surgery.
5. Incidence of postoperative nausea and vomiting (PONV).
6. Time to first ambulation.
7. Time to recovery of bowel movement.
8. Postoperative length of hospital stay.
9. Patient’s satisfaction of anesthesia and analgesia assessed by the Chinese version of Bauer questionnaire [9, 10].

### Statistical analysis

Statistical analysis was performed with SPSS 22 (SPSS, Inc., Chicago, IL, USA). Normally distributed variables were described as mean ± SD and non-normally distributed variables were described as median with interquartile range (IQR). Student’s *t* test was used for parametric data and the Mann-Whitney test for non-parametric data. Categorical data were examined using the χ 2 test (or Fisher’s Exact test as appropriate). Time-to-event data including time to flatus, ambulation time and length of hospital stay were plotted by Kaplan-Meier curves and compared by log-rank test. *P* < 0.05 was considered significant. A statistician blinded to the patient allocation was responsible for the analysis.

## Results

**Fig. 2:**
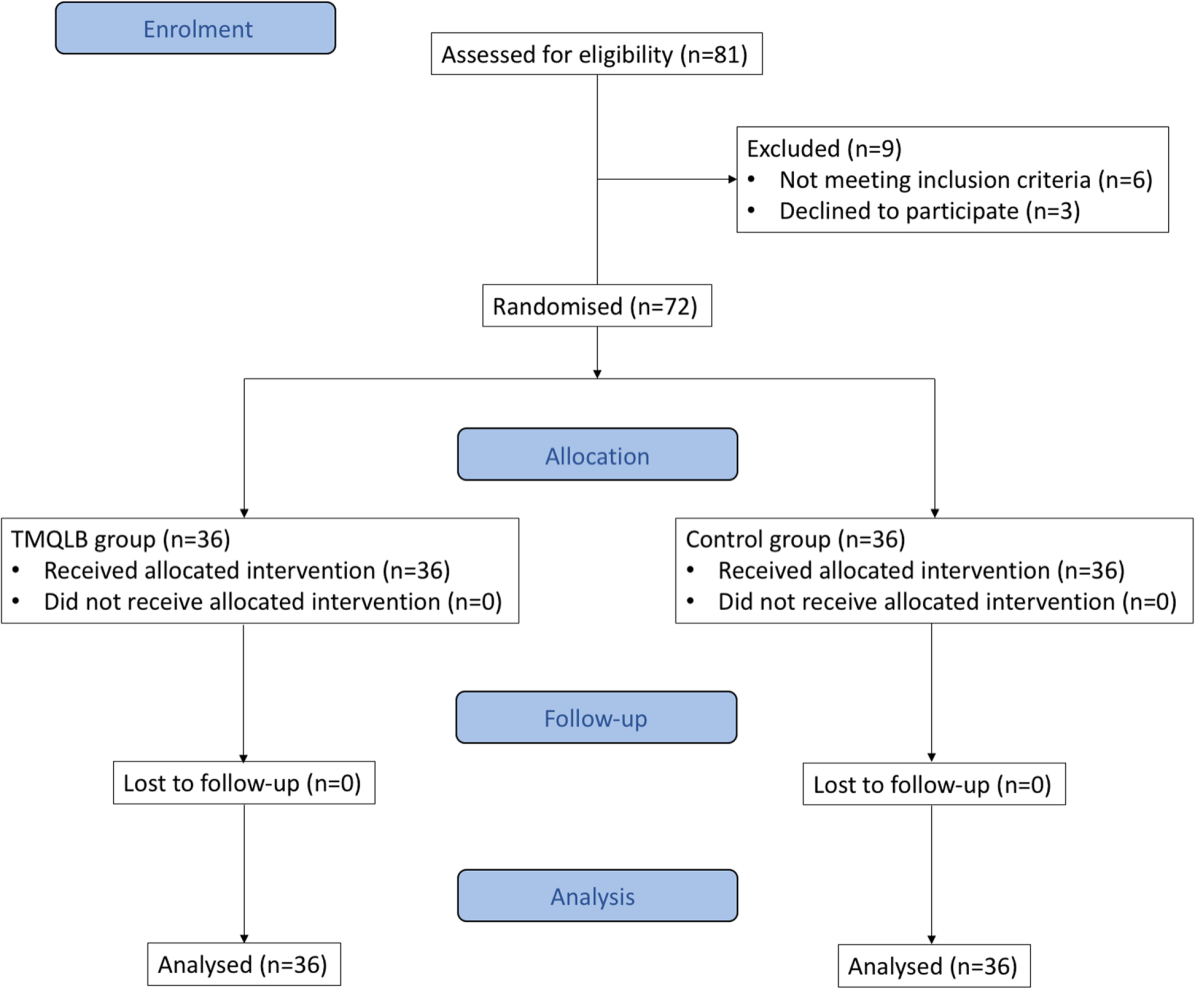
CONSORT Flow Diagram

Of the 81 patients screened, 72 patients (36 per study group) were enrolled between May 10, 2019 and September 2, 2019. Trial profile and patients’ demographics are shown in Fig. 2; Table 1, respectively. No patient dropped out of this study. The primary and secondary analgesia outcomes are shown in Table 2. Median NRS on movement at 12 h after surgery was 2 in TMQLB group (IQR 1 to 3), significantly lower than 3 of the control group (IQR 2 to 5, *p* = 0.024). NRS on movement at 2, 4 and 8 h after surgery was also significantly lower in the TMQLB group. There was no significant difference of pain at rest between these the two groups except a marginal difference at 2 h after surgery.

Intraoperative fentanyl consumption was lower in the TMQLB group compared with the control group (247.08 ± 63.54 vs. 285.44 ± 74.70, *p* = 0.022). The rate of using postoperative rescue tramadol was also significantly lower in the TMQLB group compared with the control group (5.6 vs. 27.8%, *p* = 0.027). No significant difference was observed in terms of the rate of using postoperative parecoxib in the two groups (22.2 vs. 8.3%, *p* = 0.190).

Similar incidence of nausea and vomiting was observed (11.1 vs. 25.0%, *p* = 0.220). Patients received TMQLB had better satisfaction to pain service (83.3 vs. 25.0%, *p* < 0.001), but not anesthesia service (86.1 vs. 75%, *p* = 0.234).

Time to ambulation (16.5 vs. 21 h, *p* = 0.004) and flatus (18.5 vs. 23.5 h, *p* = 0.006) were both shorter in the TMQLB group compared with control. There was no significant difference in terms of postoperative length of hospital stay between the two groups (Table 2). Regarding block-related adverse event, one patient in the TMQLB group complained newly-onset numbness and mild pain over the superior gluteal region at first day after surgery and this paresthesia lasted when he was discharged. We prescribed mecobalamine, neurotropin and pregabalin to treat his suspicious nerve injury. And the paresthesia resolved upon follow-up at one month after surgery.

**Table 1:** Demographics

Variables	TMQLB (***n*** = 36)	Control (***n*** = 36)
Age (y)	48.28 ± 11.43	47.33 ± 11.57
BMI, kg/m^2^	25.81 ± 3.91	25.14 ± 2.95
Sex		
Male	14 (38.9%)	14 (38.9%)
Female	22 (61.1%)	22 (61.1%)
ASA		
I	23 (63.9%)	24 (66.7%)
II	13 (36.1%)	12 (33.3%)
Baseline NRS	0	0
Surgery time (min)	72.42 ± 27.38	73.31 ± 20.78

Values are expressed as number (%) or mean ± SD when appropriate

Abbreviations: *BMI*, body mass index; *NRS*, numeric rating scale

## Discussion

To the best of our knowledge, this is the first prospective, randomized, triple-blind study investigating the analgesic effect of TMQLB in laparoscopic adrenalectomy. Our results show that TMQLB could significantly improve pain control on movement evaluated by NRS at 2–12 h after surgery. Moreover, the TMQLB group was associated with better patients’ satisfaction, shorter time to ambulation and flatus.

Our study has shown that the TMQLB reduced postoperative NRS on movement but not at rest. It may be because the pain at rest after laparoscopic adrenalectomy is clinically minimal and tolerable (NRS range 0–2). But the postoperative analgesia is still essential, as it facilitates early ambulation and bowel movement, corroborated by our findings, both of which are keys of enhanced recovery after surgery [11]. Our result showed statistically significant difference in NRS at 2–12 h after surgery on movement. But the clinical relevance of this result is questionable because the NRS are below 4, indicating a level of mild pain. This may be explained by the fact that rescue analgesics were prescribed once the patient experienced moderate to severe pain. However, the intraoperative fentanyl consumption and the rate of using postoperative rescue opioids were significantly lower in the TMQLB group. Therefore, we believe that TMQLB provides clinically significant analgesic effect for laparoscopic adrenalectomy.

The reduction in NRS and the opioid-sparing effect of TMQLB in laparoscopic adrenalectomy demonstrated by our study are in accordance with the findings of several studies of QLB in abdominal surgeries. Kim et al. [12] performed a meta-analysis to compare pain scores at rest and with movement in QLB and control group. There were nine RCTs of QLB in abdominal surgeries including cesarean section, laparoscopic surgeries, open liver resection and percutaneous nephrolithotomy. Pain scores in the QLB group were reduced at rest and with movement, with the most improved pain score at 12 h after surgery. Though the mean pain scores were mostly below 4, the mean differences between QLB and the control group were − 2.16 (95 % CI -3.12 to -1.20) and − 2.26 (95 % CI -3.54 to -0.98) at rest and with movement respectively. Zhu et al. [13] showed postoperative Sufentanil consumption within the first 24 h after laparoscopic nephrectomy was lower in the QLB group. And intraoperative remifentanil consumption and the number of patients requiring rescue analgesia were also lower in the QLB group. Dam et al. [14] also reported that TMQLB could reduce postoperative opioid consumption and prolong time to first opioid in laparoscopic nephrectomy.

In our study, the ambulation time was also shorter in the TMQLB group and no patient complained muscle weakness of lower extremities, indicating that no blockade of lumbar plexus occurred because weakness of the psoas, iliacus and quadriceps muscles could happen once lumbar plexus was blocked [15]. Up till now, whether or not TMQLB could block lumbar plexus remained as a conflicting issue. According to the cadaver study by Dam et al. [5], the reported analgesic effect of TMQLB in hip surgery could be attributable to local anesthetic accidentally injected into the PM muscle, which would facilitate the spread of injectate to lumbar plexus. In our study, we adopted the technique described by Dam et al. [5], aiming the needle to the fascial plane between QL and PM muscle to strictly avoid piercing the PM muscle, therefore no blockade of lumbar plexus was observed. The incidence of postoperative nausea and vomiting was not significantly different between two groups, which is likely due to our standard prophylactic use of antiemetics.

TMQLB is a safe procedure in current study without major complications, and only one patient experienced paresthesia of superior gluteal region. Considering the patient’s symptom and response to treatment, we deem this paresthesia was probably due to injury of superior cluneal nerves (SCNs), the posterior cutaneous branches of the dorsal rami of L1–3. These branches perforate the erector spinae muscle, pierce the posterior layer of the thoracolumbar fascia to become cutaneous, descend and cross the iliac crest, and innervate the skin and subcutaneous tissues of the superior gluteal region [16, 17]. Of note, a recent anatomy study demonstrated that the posterior cutaneous branch of L3 pierced the erector spinae muscle most frequently, implying greater vulnerability to injury at this level [18]. During TMQLB block, the needle pierced the erector spinae and quadratus lumborum muscle at L3 level to reach the fascia plane, which may explain the paresthesia of superior gluteal region observed.

There are some limitations of the current study. First, we used dexamethasone in the induction phase as antiemetic regimen, which may affect the assessment of pain because evidence existed that systemic dexamethasone may contribute to analgesia of regional block [19]. Second, we did not perform sensory or motor measurement because this may unblind the block allocation to the patient. Third, the study design does not allow us to further explore the dose-efficacy relationship of ropivacaine in TMQLB setting.

## Conclusions

We reported the first randomized controlled trial of TMQLB use in laparoscopic adrenalectomy. TMQLB showed better control of postoperative pain on movement with improved patients’ satisfaction of anesthesia, shorter time to ambulation and flatus.

**Table 2:** Primary and secondary outcomes

	TMQLB (***n*** = 36)	Control (***n*** = 36)	***P*** value
Primary outcome			
NRS on movement at 12 hours after surgery	2 (1-3)	3 (2-5)	0.024*
Secondary outcomes			
Intraoperative fentanyl consumption (μg)	247.08 ± 63.54	285.44 ± 74.70	0.022*
Postoperative use of tramadol	2 (5.6%)	10 (27.7%)	0.027*
Postoperative use of parecoxib	8 (22.2%)	3 (8.3%)	0.190
NRS at rest at 2 hours after surgery	2 (0-2.5)	2 (2-3)	0.037*
NRS at rest at 4 hours after surgery	2 (0-2.5)	2 (1.5-4)	0.073
NRS at rest at 8 hours after surgery	2 (1-2)	2 (1.5-3.5)	0.216
NRS at rest at 12 hours after surgery	2 (0-2.5)	2 (1-2.5)	0.360
NRS at rest at 24 hours after surgery	1 (0-2)	2 (0-2)	0.203
NRS at rest at 48 hours after surgery	0 (0-1)	1 (0-2)	0.152
NRS at rest at 72 hours after surgery	0 (0-1)	0 (0-1)	0.196
NRS on movement at 2 hours after surgery	2 (0-3)	4 (3-5)	0.001*
NRS on movement at 4 hours after surgery	2 (0.5-3)	3 (2-6)	0.003*
NRS on movement at 8 hours after surgery	3 (2-3)	3 (2-6)	0.045*
NRS on movement at 24 hours after surgery	2.5 (1-4)	3 (2-4)	0.549
NRS on movement at 48 hours after surgery	2 (1-3)	2 (2-3)	0.389
NRS on movement at 72 hours after surgery	1 (0-2)	1.5 (0-3)	0.476
Nausea and vomiting	4 (11.1%)	9 (25.0%)	0.220
Patient Satisfaction of Pain Service	30 (83.3%)	9 (25.0%)	0.000*
Patient Satisfaction of Anesthesia Service	31 (86.1%)	27 (75.0%)	0.234
Time to recovery of bowel movement (hours)	18.5 (95% CI 16.30 to 20.70)	23.5 (95% CI 19.09 to 27.91)	0.006*
Ambulation time (hours)	16.5 (95% CI 14.15 to 18.85)	21.0 (95% CI 19.04 to 22.96)	0.004*
Length of hospital stay (days)	4.0 (95% CI 3.33 to 4.67)	5.0 (95% CI 4.59 to 5.41)	0.260

Values are expressed as median (IQR), mean ± SD, number (%) or median survival (95% CI) when appropriate. *P < 0.05

Abbreviations: *NRS*, numeric rating scale

## Data Availability

The data supporting the findings of the current study are available from the corresponding author on reasonable request.

## Abbreviations

TMQLB: transmuscular quadratus lumborum block
NRS: numeric rating scale
PM: psoas major
QL: quadratus lumborum
RCT: randomized controlled trial
PONV: postoperative nausea and vomiting
IQR: interquartile range
TPVB: thoracic paravertebral block
SCN: superior cluneal nerve
ES: erector spinae
TP: transverse process
VB: vertebral body
